# Personality in Capuchin Monkeys (*Sapajus apella*): Comparison of Assessment Methods and Constructs

**DOI:** 10.1002/ajp.70214

**Published:** 2026-10-06

**Authors:** Annika Paukner, Lauren J. Wooddell, Aurelie Jolivald

**Affiliations:** ^1^ Nottingham Institute of Psychology Nottingham Trent University Nottingham UK; ^2^ Department of Neurosurgery Emory University Atlanta Georgia USA; ^3^ School of Animal, Rural & Environmental Science Nottingham Trent University Southwell UK

**Keywords:** individual differences, novel object test, observations, personality, primate, questionnaires

## Abstract

Understanding personality in nonhuman primates is critical for advancing theories of individual differences, yet methodological variation remains a major challenge. We examined whether three commonly used approaches—questionnaire ratings, behavioral observations, and experimental tests—capture stable and overlapping personality constructs in 26 captive tufted capuchin monkeys (*Sapajus apella*). Using Bayesian multivariate mixed models, we retained individual variability to assess temporal stability, cross‐context consistency, and inter‐method convergence. Behavioral observations revealed conceptually coherent clusters related to stress, activity, and social engagement, but temporal stability was low. Novelty Tests produced associations suggestive of approach–avoidance and activity–focus dimensions, yet these clusters also lacked temporal stability. Questionnaire ratings showed partial convergence with observational data but not with novelty test data. Out of 39 predicted associations, only 4 were confirmed, highlighting weak cross‐method consistency. Housing conditions significantly influenced personality expression, whereas sex and age effects were minimal. These findings suggest that personality expression may be more context‐sensitive and multidimensional than is captured by any single assessment method, and that divergence across methods may reflect different aspects of personality rather than measurement error. Integrative, multi‐method, and longitudinal designs that incorporate multiple measures may help improve the interpretation of primate personality assessments.

## Highlights

- Capuchin behavioral observations revealed coherent stress‐related, activity‐related, and social associations, but most weakened after accounting for individual and housing characteristics.
- Novelty‐test responses showed limited stability and did not converge with questionnaire ratings, indicating that these methods may capture different aspects of personality expression.
- Only 4 of 39 predicted associations were supported, demonstrating limited convergence among questionnaire ratings, naturalistic observations, and experimental tests.
- Housing condition was associated with personality expression across assessment methods, highlighting the importance of social and environmental context in primate personality research.

## Graphical abstract

Using behavioral observations, novelty tests, and questionnaire ratings, we found limited cross‐method agreement and weak temporal stability in capuchin personality. Housing conditions significantly influenced personality expression, highlighting the context‐sensitive and multidimensional nature of primate personality.

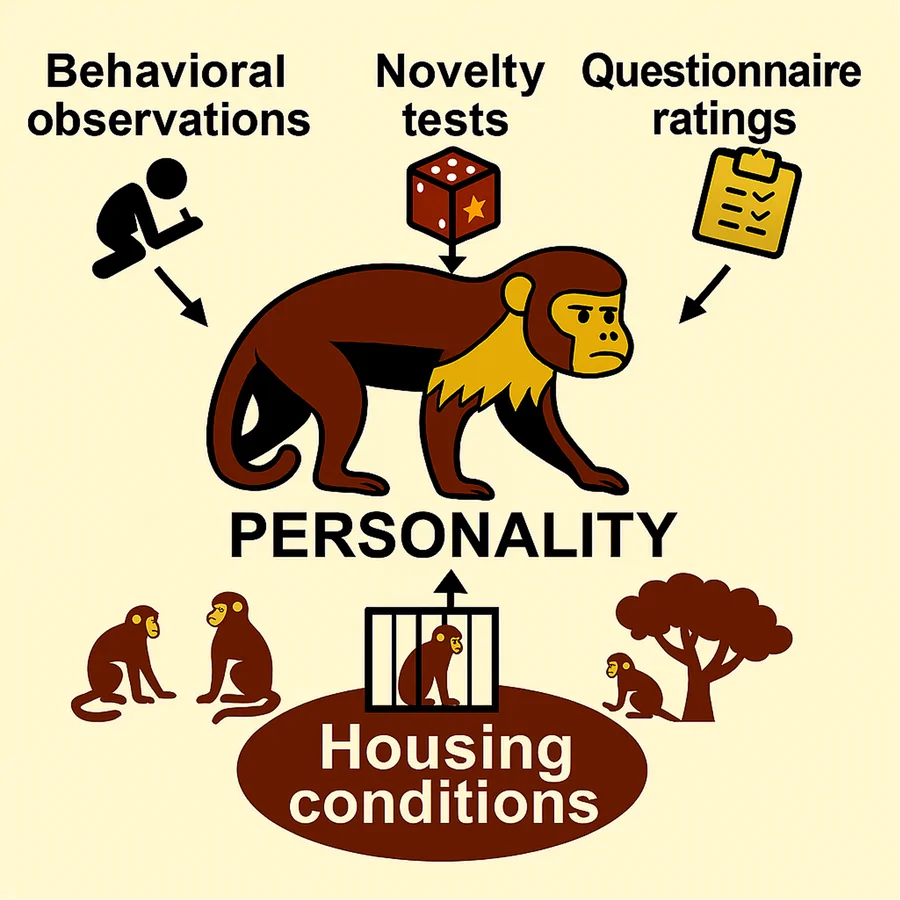

## 1 Introduction

Personality research has seen an increased interest in conceptual integration, such as combining the expression of personality traits with the biological and environmental factors that drive them, considering between‐ and within‐individual mechanisms of variation, as well as integrating human and animal personality research (Del Giudice 2022). Animal personality research is particularly suitable to shed light on the biological mechanisms and environmental factors that influence personality traits (Cavigelli 2005; Mehta and Gosling 2008; Stamps and Groothuis 2010; Trillmich et al. 2018), offering insights into the evolution and ecology of personality in different species (Dall and Griffith 2014). However, animal personality research has its own conceptual issues. For example, there are ongoing discussions about the extent to which animal personality is a genuine and consistent trait, and whether certain species and taxa are even capable of possessing personality traits (Kaiser and Müller 2021). Due to ecological and social differences between species, the question of whether animal personality can be reliably compared across species remains a major challenge in the field (White et al. 2020).

For a behavioral trait to be considered a personality trait, it must demonstrate both temporal stability and contextual consistency, meaning that an individual's behavior should reliably manifest over time and across different situations (Kaiser and Müller 2021). However, temporal stability and contextual consistency do not imply that personality traits are expressed identically in all situations or throughout the lifespan. Research in both human and animal personality has increasingly recognized that stable individual differences can co‐exist with context dependent variation in behavior (Stamps and Groothuis 2010; van Oers et al. 2005). Environmental and social conditions, developmental changes, and life experiences may influence how personality traits are expressed without necessarily altering the underlying trait structure (Stamps and Groothuis 2010; Del Giudice 2022). Consequently, personality is often viewed not as a fixed and inflexible characteristic, but as a dynamic system in which stable dispositions interact with situational influences to produce behavior.

In addition to conceptual issues, there are also methodological challenges to studying animal personality. Several different methods have been used to assess personality in animals, including experimental tests (such as maze tests [e.g., Campbell et al. 2021], social interaction tests [e.g., Tõnissaar et al. 2004], open field tests [e.g., Perals et al. 2017], novel object tests [e.g., Takola et al. 2021]), questionnaire studies, and observations of naturalistic, everyday behaviors. Yet when different methods are applied to the same animals to confirm contextual consistency, the results are often mixed. While multiple methods of personality assessment fare quite well in great apes (e.g., Padrell et al. 2020; Uher and Asendorpf 2008; Freeman et al. 2013), less concordance between assessment methods has been reported in other primates. For example, Carter et al. (2012) used two different experimental assessments of the construct “boldness” in chacma baboons (*Papio ursinus*) and did not find significant correlations between the two measurements. Tkaczynski et al. (2019) used an experimental test, observer ratings, and behavioral observations in Barbary macaques (*Macaca sylvanus*) and reported low agreement between these methods. Šlipogor et al. (2020) compared the personality structure of common marmosets (*Callithrix jacchus*) derived from an experimental test, observer ratings, and behavioral observations, and found that the observer ratings did not correlate with observed behavior (see also Iwanicki and Lehmann 2015). Together, these findings suggest that cross‐method divergence may reflect differences in the constructs measured, the contexts sampled, or the sources of measurement error associated with each approach (Freeman et al. 2011). Similarly, the temporal stability of animal personality traits has seen mixed results. While some have reported stability of constructs over time (chimpanzees [*Pan troglodytes*]: Koski 2011; rhesus macaques [*Macaca mulatta*]: Capitanio 1999; marmosets: Šlipogor et al. 2021), others have found that personality expression can vary across time and can be affected by factors such as social group composition or other environmental influences (chimpanzees: Rawlings et al. 2020; rhesus macaques: von Borell et al. 2016).

These mixed findings raise a further question: how much temporal and contextual sampling is required to characterize personality reliably? Different assessment methods capture behavior at different temporal scales and under different environmental conditions. For example, questionnaire ratings often integrate observations accumulated over long periods and across many situations, whereas behavioral observations and experimental tests typically sample behavior within narrower time windows and contexts. Consequently, apparent differences in stability across methods may reflect differences in sampling resolution rather than differences in the underlying personality constructs being measured. Determining the amount of temporal and contextual information required to characterize personality remains an important challenge in animal personality research.

Given these conceptual and methodological issues, finding stable personality traits and comparing them across contexts and species is made more difficult by the fact that it is still unclear which personality assessment techniques produce the most valid and reliable results. In the present study, which is a retrospective analysis of existing datasets, we used behavioral observations, questionnaire ratings, and an experimental assessment to evaluate personality measures in tufted capuchin monkeys (*Sapajus apella*) across time and context. The three approaches used here sampled behavior over different temporal and contextual scales, enabling us to examine their convergence. Capuchin monkeys are an excellent model species in this respect, as past research has shown stability of some, but not all, personality traits across different assessment methods (Morton et al. 2013; Fernández‐Bolaños et al. 2020; Uher et al. 2013). Contributing to these mixed results may have been the data reduction techniques that researchers have used. Although reducing complex behavioral datasets is often necessary, approaches that aggregate repeated observations into individual‐level summary measures before conducting principal component or correlational analyses can obscure within‐individual variation across time and context and may not fully carry measurement uncertainty into subsequent analyses (Houslay and Wilson 2017). In the present study, we used multivariate mixed‐effect models that incorporated repeated observations directly, without summary, allowing within‐ and between‐variation to be estimated simultaneously and thus providing a more detailed assessment of personality expression across time, context, and measurement method. Given the retrospective design and modest sample, our aim was to explore whether the methods capture overlapping or distinct aspects of personality expression and to identify considerations for future multi‐method research.

## 2 Methods

All data were collected at the Laboratory of Comparative Ethology, National Institutes of Health Animal Center, near Poolesville, Maryland, USA. All applicable national and institutional guidelines for the care and use of animals were followed. All procedures adhered to the American Society of Primatologists (ASP) Principles for the Ethical Treatment of Non‐Human Primates and complied with protocols approved by the *Eunice Kennedy Shriver* National Institute of Child Health and Human Development Animal Care and Use Committee, ASP #12‐015 and #15‐015.

### 2.1 Subjects

Subjects were 26 tufted capuchin monkeys (*S. apella*), 9 adult males and 9 adult females (5+ years old at the beginning of the study in 2012), as well as 7 juvenile males and 1 juvenile female (2–4 years old at the beginning of the study in 2012). All animals were captive‐born and housed at the National Institute of Health Animal Center in Maryland, USA. Seven animals were housed in small groups of 2–3 animals in cages (1.78 m × 0.76 m × 1.85 m) with continuous full contact (pair/group); the remaining 19 animals were part of two larger breeding groups that were housed in indoor runs during the winter (1–2 connected runs, each 2.3 m × 4.1 m × 2.1 m) and in large outdoor enclosures during the summer (ca. 250 m^3^) with continuous full contact (group). All enclosures were enriched with perches and/or swings and various plastic manipulanda. Monkeys were not food‐deprived and received daily provisions of seeds and fresh fruits or nuts. Water and commercial monkey biscuits were available *ad libitum*.

### 2.2 Procedure

#### 2.2.1 Behavioral Observations (Data Collected in 2012)

A trained observer collected the data over a 6‐week period. Each monkey was individually observed within their social group for 5 min, twice per week, using the ethogram below (Table 1). However, because the following behaviors were either never observed or occurred only very rarely, they were unsuitable for analysis and therefore excluded: Passive, Social Play, Social Groom (Receive and Initiate), Aggression (Receive and Initiate), Urine Wash, Submission (Receive and Initiate), Mount (Receive and Initiate), and Yawn.

**Table 1:** Ethogram used during Behavioral Observations.

Variable	Definition
Pacing*	Repetitive, undirected walking, running, or climbing, typically in a recognizable pattern. Measured in seconds.
Physical exploration*	Manipulation of objects or enclosure by hand and/or mouth. Measured in seconds.
Visual explore*	Monkey actively scans the environment while stationary. Measured in seconds.
Locomotion*	Directed movement from one location to another. Measured in seconds.
Social groom (received or initiated)*	Sweeping the fur of another individual with one hand, interspersed with plucking movements by the other hand and/or mouth. Measured in seconds.
Passive*	Monkey sits or lies in a relaxed/drowsy manner; includes sleeping. Measured in seconds.
Social Play*	Playful interaction with other individuals that may include wrestling or chasing, with or without play faces. Measured in seconds.
Self‐groom*	Sweeping own fur with one hand, interspersed with plucking movements by the other hand and/or mouth. Measured in seconds.
Social	Monkey is within arm's reach (ca. 30 cm) of another individual. Measured in seconds.
Aggression (received or initiated)	Monkey displaces, threatens, chases, or physically attacks another individual. Measured as frequency.
Scratch	Common usage. Measured as frequency.
Urine wash	While standing quadrupedally, the monkey urinates into the palm of one hand and rubs it on the sole of the ipsilateral foot. Measured as frequency.
Head toss	Twisting or twirling the head in an exaggerated way. Measured as frequency.
Submission (received or initiated)	Displacement by another individual, or fear grimace and/or fear vocalizations directed at another individual. Measured as frequency.
Mount (received or initiated)	Ventro‐dorsal contact between two individuals. Measured as frequency.
Vocalization	Includes any vocalization made by the individual. Measured as frequency.
Yawn	Common usage. Measured as frequency.

*Note:* Behaviors marked with * were mutually exclusive. The variable Social could co‐occur with physical exploration, visual explore, passive, and self‐groom, but not pacing, locomotion, social play, or social groom.

#### 2.2.2 Novelty Tests (Data Collected in 2012 and 2015)

The Novelty Test was based on procedures published by Coleman et al. (2011). Subjects were temporally separated from their social group and given a few minutes to acclimatize to the test cage before the formal assessment began. While in the test cage, subjects could see and hear (but not touch) members of their social group. All tests were video‐taped and coded off‐line.

The test began by placing a novel food (a slice of kiwi) on a platform just outside the test cage; monkeys could reach through a gap in the test cage to access the food. Once the food was placed, the experimenter left the room. After 5 min, the experimenter returned, removed any remaining pieces of food from the platform, placed a brightly colored rubber toy on the platform, and then left the room. After 5 min, the experimenter returned, removed the toy, and placed a small rubber snake (ca. 30 cm long) on the platform close to the test cage. Half an apple (a preferred food item) was placed behind the snake so that monkeys had to reach over the snake to access the apple. All monkeys had previously been housed in outdoor enclosures where native snake species were occasionally encountered, and animal care and research staff had observed alarm and threat responses directed towards snakes, suggesting that snakes represent an ecologically relevant and potentially threatening stimulus for this population. The experimenter left the room again and returned after 5 min, which concluded the test, and subjects were reunited with their social group. This test (novel food, novel object, novel threat) was performed twice, once in 2012 and once in 2015.

#### 2.2.3 Personality Ratings (Data Collected in 2012)

The Hominid Personality Questionnaire (HPQ; Weiss et al. 2009) was used to provide a personality rating for each monkey. Two raters who were familiar with the monkeys independently rated each monkey on 54 items on a 7‐point scale with 1 indicating total absence of a trait and 7 indicating extremely large amounts of a trait (e.g., playful, submissive, excitable, anxious, etc.).

### 2.3 Data Coding

For each condition of the Novelty Test, we measured the latency to touch the food/toy/apple (in seconds) as well as the number of touches to the object over the 5‐min assessment periods. In addition, we measured the duration of six behaviors as well as the frequency of seven behaviors (Coleman et al. 2011). However, six of the behaviors (freeze, sleep, stereotypical behaviors, lipsmack, teeth grind, and yawn) did not occur during the tests, while vocalizations could not be clearly distinguished between being produced by the subject or by other members of the social group. This left a total of 7 behaviors for analysis (see Table 2).

**Table 2:** Behaviors scored during the Novelty Test following Coleman et al. (2011).

Variable	Definition
Touch	Number of touches to the novel food/toy/snake. Frequency.
Latency	Latency to touch the novel food/toy/snake. Measured in seconds.
Stationary	Subject sits, stands, or hangs at the side or top of the cage while monitoring their environment. Duration in seconds.
Locomotion	Subject walks/runs/climbs/jumps. Duration in seconds.
Explore object	Subject touches, manipulates, or holds the novel object. Duration in seconds.
Scratch	Common usage. Frequency.
Threat	Any threatening behavior, including bared teeth, forehead raise, and piloerection. Frequency.

*Note:* Behaviors measured as durations were mutually exclusive, while behaviors measured as frequencies could co‐occur with other behaviors.

For the HPQ, the mean for each item was computed across both raters and entered into a principal component analysis as part of a larger data set (Morton et al. 2013). Components were rotated using both varimax and promax procedures, and loadings ≥ |0.4| were defined as salient. This analysis yielded five factors: Assertiveness, Openness, Neuroticism, Sociability, and Attentiveness (for further details, see Morton et al. 2013).

### 2.4 Data Analysis

All data analysis was carried out in R 4.2.2. (R Core Team 2022). Throughout, Bayesian multivariate linear mixed models (mLMMs) were fitted using the *MCMCglmm* package (Hadfield 2010). Briefly, mLMMs allow the modeling of several response variables within the same model and can therefore estimate both the variance and the impact of fixed effects for each response, and the covariance between them (Houslay and Wilson 2017). Repeated measures of outcomes variables can be entered into an mLMM without the need for data summarization, thus allowing uncertainty to be factored into the calculation of the correlation between outcome variables (Houslay and Wilson 2017). Bayesian mLMM convergence was assessed visually from the mixing of four chains on trace plots, and through Gelman and Rubin's convergence diagnostic obtained using the *coda* package (Plummer et al. 2006). Alongside the posterior distribution of model parameters, *MCMCglmm* outputs “pMCMC” as an analog of the frequentist two‐tailed *p* value (Hadfield et al. 2013); this value is referred to thereafter in text as “*p*” and is used to identify significant effects with a threshold of *α* = 0.05.

#### 2.4.1 Models' Specifications

Because several related multivariate models were fitted to address different datasets and research questions, abbreviated model identifiers are used throughout this section for brevity. A complete overview of all models, including the data analyzed, model structure, fixed effects, and research question addressed, is provided in Table S1.

All response variables were scaled before being modeled using mLMMs. All outcome variables except personality factors were repeated measures and were therefore modeled with individual ID entered as a random effect. In addition, previous research indicates that age category, sex, and housing conditions (Rawlings et al. 2020) can affect behavioral expression and may therefore influence both the outcome variables and the correlations among them. To evaluate the impact of those potential influential factors on the outcome of personality analyses, all models were run twice: (1) first as a null model, i.e. with no fixed effects included, to mimic traditional consistency and correlation analyses which do not take confounders into account, (2) then as a full model with age category, sex and housing included as fixed effects to test whether observed associations remained once these variables were accounted for.

We fitted separate multivariate models for personality questionnaire factors (mLMM_PF), behaviors retained in the Behavioral Observations (mLMM_BO), novelty‐test data collected in 2012 (mLMM_NT_2012), novelty‐test data collected in 2015 (mLMM_NT_2015), and the combined novelty‐test dataset (mLMM_NT). Novelty type (food/object/threat) was entered as an additional fixed effect in all Novelty Test models, and Year (2012/2015) was entered in mLMM_NT. Full model outputs showing the impact of fixed effects, including age category, sex, and housing condition, on outcome variables are available in Tables S2–S4.

Finally, we fitted sets of pairwise personality‐behavior models relating questionnaire personality factors to behaviors derived from Behavioral Observations (mLMM_BOi_PFj) and to novelty‐test behaviors (mLMM_NTi_PFj). Novelty type (food/object/threat) and Year (2012/2015) were entered as additional fixed effects applied only to the Novelty Test behavior response variable in all mLMM_NTi_PFj.

#### 2.4.2 Temporal Stability of Behavioral Measures

Intra‐class correlations (ICCs) and adjusted repeatabilities (the proportion of total variance explained by individual ID once fixed effects have been accounted for in a linear mixed model [Hertel et al. 2020]) were used to quantify individual stability in behavior over repeated measures during the Behavioral Observations and Novelty Test procedures (Dingemanse and Dochtermann 2013; Hertel et al. 2020). They were derived from null and full mLMMs, respectively, along with their 95% credible intervals, using code from Houslay and Wilson (2017). To determine whether individuals' behavior was stable across Behavioral Observation sessions, the ICC and adjusted repeatability of each behavior were derived from mLMM_BO. ICCs and adjusted repeatabilities were also derived from mLMM_NT_2012, mLMM_NT_2015, and mLMM_NT to quantify (i) cross‐context consistency of responses to novel food, novel object, and novel threat in 2012, (ii) cross‐context consistency of responses to novel food, novel object, and novel threat in 2015, and (iii) temporal stability of responses to novelty between 2012 and 2015, respectively.

#### 2.4.3 Within‐Context Behavioral Syndromes During Behavioral Observations and Novelty Tests

Correlations between mLMM response variables and their 95% credible intervals were extracted from the mLMM (co)variance matrix using code from Houslay and Wilson (2017). The posterior distribution of the among‐individual correlation between two response variables is calculated by dividing the corresponding covariance between the two variables by the product of the square root of their variances (Houslay and Wilson 2017). To investigate whether within‐context behavioral syndromes, that is, patterns of inter‐correlation between behaviors, could be observed during Behavioral Observations and during Novelty Tests, pairwise mean and 95% credible intervals correlations between response variables were derived from mLMM_BO and mLMM_NT, respectively.

#### 2.4.4 Pairwise Correlations Between Observed Behavior and Personality Questionnaire Factors

To investigate whether personality questionnaire factors predicted behavior during Behavioral Observations and during Novelty Tests, pairwise mean and 95% credible intervals correlations between personality questionnaire factors and behavior were extracted from all mLMM_BOi_PFj and mLMM_NTi_PFj models, respectively. These observed correlations were compared with hypothesized correlations based on pre‐existing literature (Morton et al. 2013) as presented below in Table 3.

**Table 3:** Hypothesized correlations between personality factors and behaviors during Behavioral Observations and Novelty Tests, based partly on pre‐existing literature (Morton et al. 2013).

	Assertiveness	Openness	Neuroticism	Sociability	Attentiveness
Behavioral Observations					
Pacing	−	+	+		
Physical exploration		+			
Visual exploration		−*	−*	−*	+*
Locomotion		+			
Social	+*			+*	+
Self‐groom	−				
Scratch	−		+		
Head toss	−		+		
Vocalization			+	−	−
Novelty Testing					
Touch	+	+	+		−
Latency	−	−	−		+
Stationary		−	−		
Locomotion		+	+		
Explore		+	+		
Scratch	−		+		
Threat	+		+	−	

*Note:* + denotes a significantly positive correlation is hypothesized and − denotes a significantly negative correlation, while empty cells denote that no significant correlation is hypothesized. Cells marked with * denote hypotheses where the personality questionnaire factor has previously been validated against behavior (Morton et al. 2013).

## 3 Results

### 3.1 Impact of Age, Sex, and Housing Condition on Behavior and Personality

During Behavioral Observations, Vocalization was the only behavior significantly predicted by Age and Sex: juveniles were significantly less likely to vocalize than adults (posterior mean [95% CI] = −0.693 [−1.329; −0.056], *p* = 0.036), while males were significantly more likely to vocalize than females (posterior mean [95% CI] = 0.692 [0.08; 1.292], *p* = 0.031). Housing conditions had a strong impact on behavior. Compared to individuals housed in breeding groups, pair‐housed individuals were significantly more likely to pace (posterior mean [95% CI] = 1.647 [0.883; 2.384], *p* < 0.001) and self‐groom (posterior mean [95% CI] = 0.764 [0.027; 1.435], *p* = 0.034), and had a non‐significant tendency to head toss more often (posterior mean [95% CI] = 1.243 [−0.261; 2.734], *p* = 0.099). In contrast, they spent significantly less time in social proximity (posterior mean [95% CI] = −1.111 [−2.06; −0.204], *p* = 0.024) and visual exploration (posterior mean [95% CI] = −0.975 [−1.866; −0.020], *p* = 0.044) and vocalized significantly less (posterior mean [95% CI] = −0.825 [−1.526; −0.165], *p* = 0.020) than individuals in the breeding groups.

During Novelty Tests, Age and Sex had no significant effect on any of the behaviors recorded. However, pair‐housed individuals spent significantly more time exploring the novel objects (posterior mean [95% CI] = 0.583 [0.021; 1.134], *p* = 0.048) and less time in locomotion (posterior mean [95% CI] = −1.145 [−1.761; −0.515], *p* < 0.001), and showed a non‐significant tendency to scratch more often than breeding group individuals (posterior mean [95% CI] = 0.690 [−0.076; 1.466], *p* = 0.073). Novel Object type also significantly predicted both interactions with the novel object: total time spent exploring (novel food vs. novel object: posterior mean [95% CI] = −0.889 [−1.233; −0.513], *p* < 0.001; novel food vs. novel threat: posterior mean [95% CI] = −1.246 [−1.617; −0.87], *p* < 0.001), latency to first touch (novel food vs. novel threat: posterior mean [95% CI] = 1.051 [0.636; 1.432], *p* < 0.001), and frequency of touches (novel food vs. novel object: posterior mean [95% CI] = 0.476 [0.134; 0.808], *p* = 0.008). Novel Object type also significantly impacted behavior during the Novelty Tests: time spent stationary (novel food vs. novel object: posterior mean [95% CI] = 0.943 [0.54; 1.327], *p* < 0.001; novel food vs. novel threat: posterior mean [95% CI] = 1.076 [0.681; 1.49], *p* < 0.001), frequency of scratching (novel food vs. novel object: posterior mean [95% CI] = 0.500 [0.139; 0.886], *p* = 0.013), and frequency of threatening behavior (novel food vs. novel threat: posterior mean [95% CI] = 0.897 [0.497; 1.344], *p* < 0.001).

Sex did not significantly predict any of the personality questionnaire factors, and there was only a non‐significant tendency for Age to predict Assertiveness (lower Assertiveness in juveniles, posterior mean [95% CI] = −1.381 [−2.908; 0.180], *p* = 0.081) and Openness (higher Openness in juveniles, posterior mean [95% CI] = 1.307 [−0.046; 2.648], *p* = 0.062). Housing condition significantly predicted Neuroticism and Attentiveness: compared to breeding group individuals, pair‐housed individuals were significantly more Neurotic (posterior mean [95% CI] = 1.581 [0.330; 2.829], *p* = 0.019) and significantly less Attentive (posterior mean [95% CI] = −1.497 [−2.532; −0.479], *p* = 0.010).

### 3.2 Repeatability of Behavioral Measures

During Behavioral Observations, the proportion of variance explained by individual ID was low but significantly different from 0 for most behaviors (Figure 1a). However, Pacing and Head Toss, two stereotypic behaviors, were highly repeatable. Adjusted repeatabilities were much lower than the ICCs for all behaviors (Figure 1a), indicating that shared individual characteristics such as age, sex, and housing condition contributed to artificially increasing ICCs.

**Figure 1:**
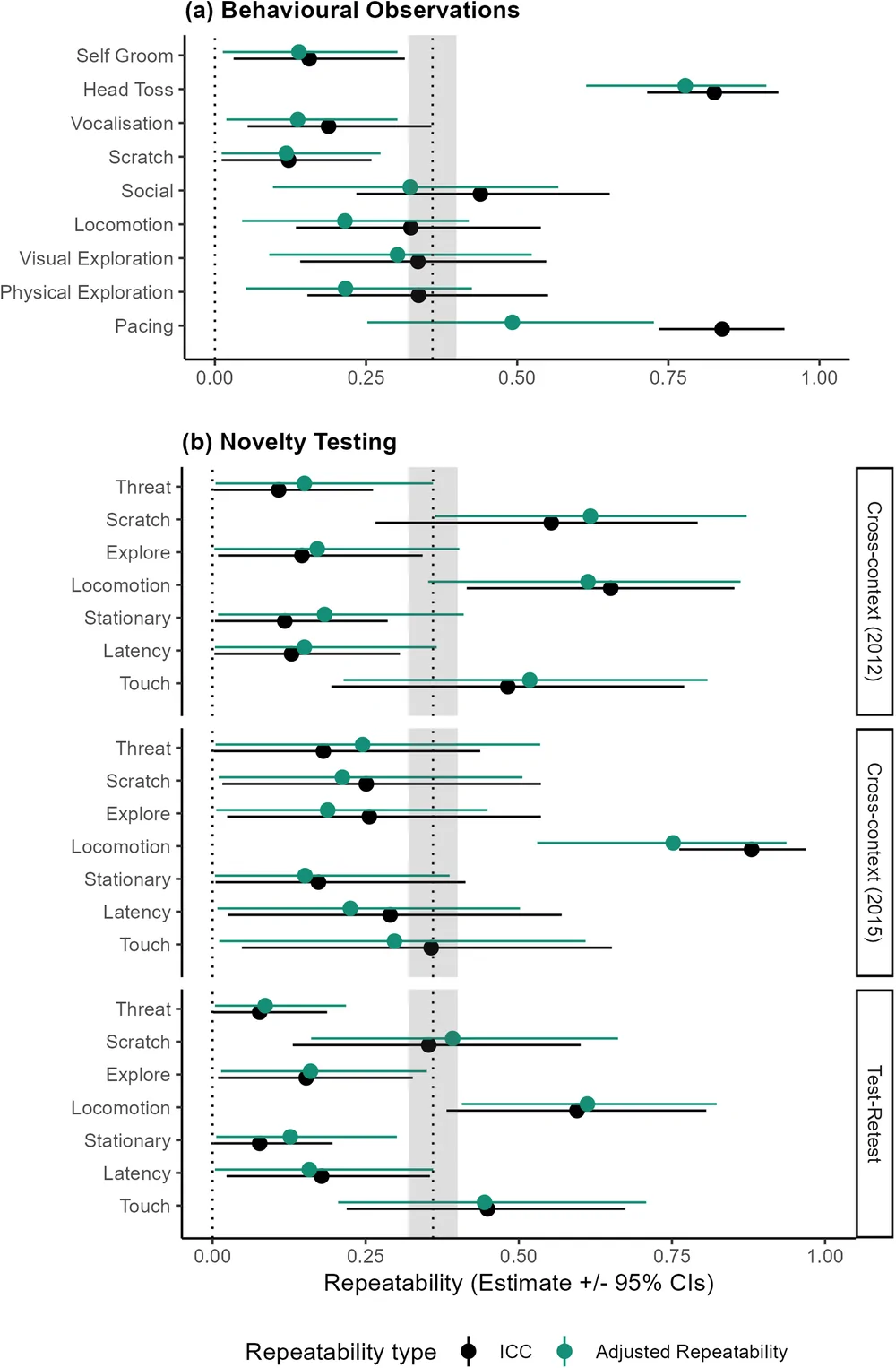
Estimates and 95% credible intervals of ICC and adjusted repeatability for all behaviors during (a) Behavioral Observations and (b) Novelty Tests. The dashed line and grayed area represent the estimate and 95% credible interval for the mean repeatability of animal behavior derived in a meta‐analysis (Bell et al. 2009) and are included as a benchmark to facilitate interpretation of the repeatabilities in this study.

During Novelty Testing, the repeatability of latency to explore the novel objects, time spent exploring the novel objects, time spent stationary, and threatening behavior was low both across contexts and time (Figure 1b). However, time spent on locomotion showed high temporal stability and high cross‐context consistency in both 2012 and 2015. The frequency of scratching and touching the novel object also showed high temporal stability, and high cross‐context consistency in 2012, but not in 2015 (Figure 1b). Adjusted repeatabilities were close to or higher than ICCs (Figure 1b).

### 3.3 Within‐Context Behavioral Syndromes During Behavioral Observations and Novelty Tests

#### 3.3.1 Behavioral Observations

The null model yielded several patterns of credible pairwise correlations. Pacing, Head tossing, and Self‐grooming were strongly positively intercorrelated (Pacing/Head toss: *r̄* [95% CI] = 0.768 [0.531; 0.955]; Pacing/Self‐groom: *r̄* [95% CI] = 0.583 [0.078; 0.953]; Head toss/Self‐groom: *r̄* [95% CI] = 0.634 [0.139; 0.98]). However, Scratch, another self‐directed behavior, was not associated with this cluster. Locomotion and Physical exploration were strongly positively correlated (*r̄* [95% CI] = 0.684 [0.273; 0.988]), and were both negatively associated with the cluster of stereotypic and self‐directed behavior (Pacing/Locomotion: *r̄* [95% CI] = −0.701 [−0.976; −0.36]; Pacing/Physical exploration: *r̄* [95% CI] = −0.825 [−0.987; −0.581]; Head toss/Locomotion: *r̄* [95% CI] = −0.618 [−0.948; −0.214]; Head toss/Physical exploration: *r̄* [95% CI] = −0.61 [−0.915; −0.208]; Locomotion/Self‐groom: *r̄* [95% CI] = −0.595 [−0.976; −0.061]). Finally, Social proximity, Vocalizations, and Visual exploration formed a third cluster of strongly positively intercorrelated behaviors (Social/Vocalization: *r̄* [95% CI] = 0.586 [0.099; 0.958]; Social/Visual Exploration: *r̄* [95% CI] = 0.598 [0.173; 0.94]; Vocalization/Visual Exploration: *r̄* [95% CI] = 0.801 [0.474; 0.998]). Pacing was significantly negatively correlated with Social proximity (*r̄* [95% CI] = −0.634 [−0.924; −0.252]), but there were no other intercorrelations between the behaviors in both clusters.

When age, sex, and housing condition were incorporated into the full model, the relationships detected by the null model weakened such that most were no longer significant. Only the positive correlation between Pacing and Head tossing (*r̄* [95% CI] = 0.57 [0.123; 0.933]), the negative correlation between Pacing and Physical exploration (*r̄* [95% CI] = −0.629 [−0.983; −0.106]), and the positive correlation between Vocalization and Visual exploration (*r̄* [95% CI] = 0.654 [0.094; 0.991]) remained significant.

#### 3.3.2 Novelty Testing

The null model showed few patterns of intercorrelation between behaviors during Novelty Testing. The number of touches to the novel object was significantly positively correlated with the frequency of scratching (*r̄* [95% CI] = 0.627 [0.177; 0.965]), and significantly negatively correlated with the latency to first touch the novel object (*r̄* [95% CI] = −0.775 [−0.997; −0.357]). In addition, time spent in locomotion was significantly negatively correlated with the time spent exploring the novel object (*r̄* [95% CI] = −0.822 [−0.999; −0.445]).

When age, sex, and housing condition were incorporated into the full model, only the positive correlation between the number of touches to the novel object and the frequency of scratching remained significant (*r̄* [95% CI] = 0.527 [0.004; 0.95]).

### 3.4 Pairwise Correlations Between Observed Behavior and Personality Questionnaire Factors

#### 3.4.1 Behavioral Observations

Assertiveness was significantly positively correlated with Locomotion during the Behavioral Observations (*r̄* [95% CI] = 0.518 [0.043; 0.996]) and negatively correlated with Scratching (*r̄* [95% CI] = −0.751 [−0.998; −0.226]). Openness was positively correlated with Physical exploration (*r̄* [95% CI] = 0.509 [0.001; 0.996]) and negatively correlated with Scratching (*r̄* [95% CI] = −0.78 [−0.998; −0.268]). Neuroticism was strongly positively correlated with Vocalizations (*r̄* [95% CI] = 0.818 [0.335; 0.999]). Sociability was negatively correlated with Scratching (*r̄* [95% CI] = −0.655 [−0.998; −0.021]); in addition, a negative correlation with Vocalizations was very close to significance (*r̄* [95% CI] = −0.716 [−0.997; 0.008]). Finally, Attentiveness was negatively correlated with Social proximity (*r̄* [95% CI] = −0.48 [−0.863; −0.015]) and with Vocalizations (*r̄* [95% CI] = −0.823 [−0.998; −0.367]). No other correlations between behaviors during Behavioral Observations and personality questionnaire factors were significant (Figure 2a).

**Figure 2:**
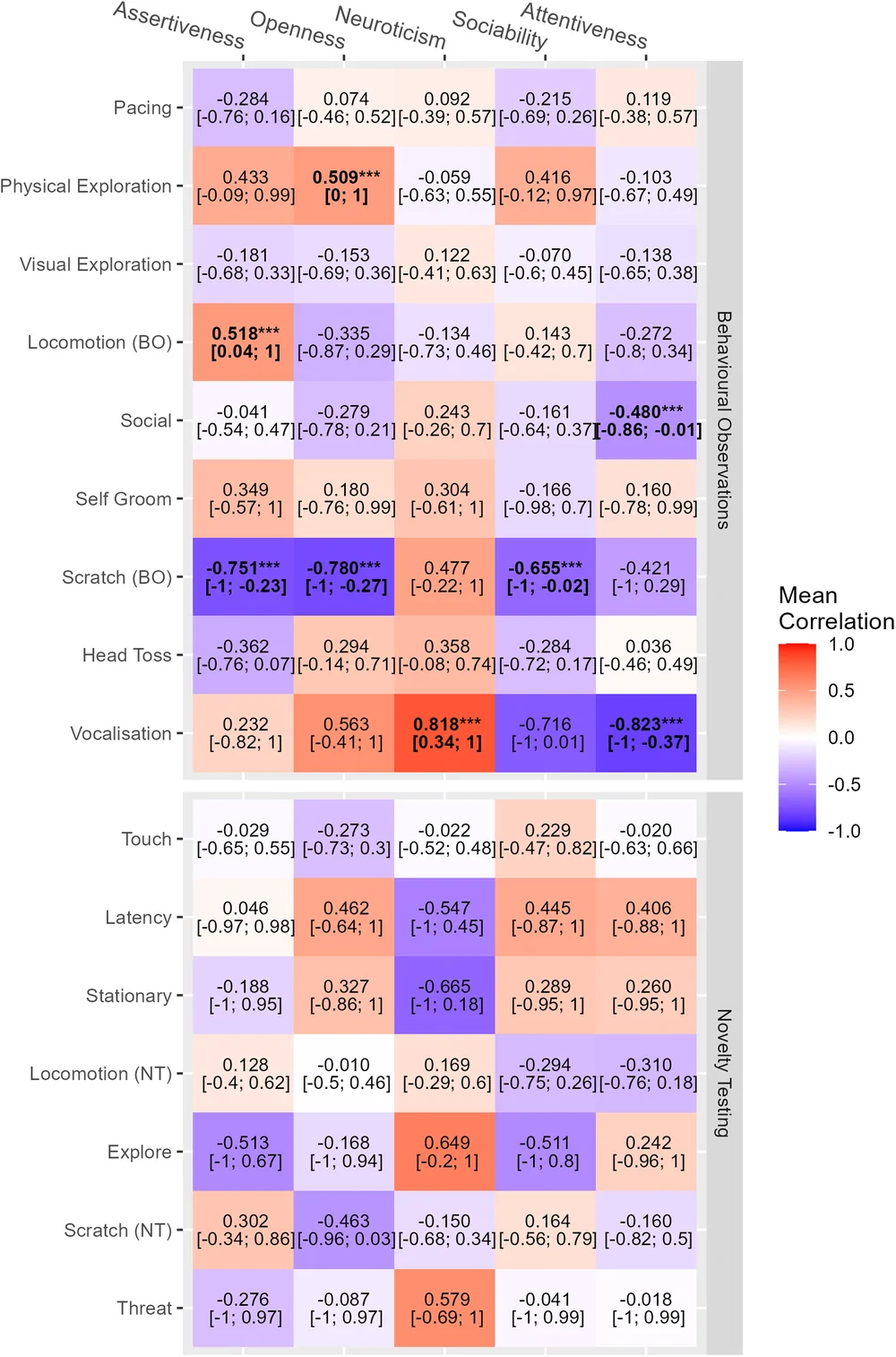
Posterior mean and 95% credible interval estimate of pairwise correlations between observed behavior during (a) Behavioral Observations and (b) Novelty Testing and ratings on personality factors derived from the Hominid Personality Questionnaire (2012). “***denotes significant correlations (0 not in the 95% credible interval for the correlation coefficient)”.

#### 3.4.2 Novelty Testing

There were no significant correlations between behaviors during Novelty Testing and personality questionnaire factors derived from the HPQ (Figure 2b).

## 4 Discussion

The present study set out to examine whether commonly used assessment tools capture stable individual differences, context‐dependent behavioral expression, or a combination of both. We focused on tufted capuchin monkeys and examined three commonly used approaches (questionnaire ratings, behavioral observations, and experimental tests) in the same individuals with the expectation that these methods would converge on shared dimensions of personality by demonstrating temporal stability and cross‐contextual consistency. Employing Bayesian multivariate mixed models, we aimed to preserve individual variability and avoid data reduction techniques that might obscure meaningful patterns. Overall, Behavioral Observations and Novelty Tests yielded conceptually coherent associations, but evidence of stability over the sampled periods and contexts was limited. Questionnaire ratings showed partial convergence with naturalistic observations but not with novelty‐test responses, while housing condition was associated with substantial variation across several measures. These findings should be considered while taking into account the different temporal scales and contexts of the assessments.

Models that did not account for age, sex, and housing condition revealed three coherent clusters in the Behavioral Observation data that align well with established frameworks of primate behavior. The first cluster comprised pacing, head tossing, and self‐grooming, behaviors typically classified as stereotypic or self‐directed and often associated with stress (Mason 1991; Maestripieri et al. 1992). The second cluster included locomotion and physical exploration, traits frequently linked to dimensions such as activity or exploration in animal personality research (Réale et al. 2007). The third cluster included social proximity, vocalizations, and visual exploration, suggesting a domain related to sociability, which is one of the most consistently identified personality dimensions across primate species (Freeman and Gosling 2010). Conceptually, these clusters represent distinct behavioral domains (stress‐related, activity‐related, and social) rather than arbitrary groupings. However, despite this internal coherence, these clusters were not maintained once the additional factors of age, sex, and housing conditions were accounted for. In addition, low adjusted repeatabilities suggest that temporal stability across observation periods was limited when age, sex, and housing conditions were controlled, echoing previous findings that short observation periods produce low repeatability of behavior (Bell et al. 2009), which may reflect context‐dependent expression, insufficient sampling, or both, rather than the absence of underlying stable individual differences. This result suggests that while behavioral clusters are meaningful within a certain context, they do not provide strong evidence for temporal stability over the observation period sampled here and should therefore be interpreted cautiously as enduring personality constructs (Kaiser and Müller 2021).

In models that did not account for age, sex, and housing condition, novelty test behaviors revealed a set of associations that suggest underlying dimensions of responses to unfamiliar stimuli. Individuals who touched the novel object more frequently also exhibited shorter latencies before first contact and higher scratching rates, a combination that may reflect an ambivalent response of both curiosity and arousal. In contrast, time spent in locomotion was negatively associated with time spent exploring the object, suggesting a trade‐off between general activity and focused engagement. However, as was the case with behavioral observations, these clusters were not maintained in models that accounted for age, sex, and housing conditions. Stability across time was low, with only locomotion, frequency of scratching, and touching the novel object retaining stability. While this pattern may indicate that these behavioral clusters are influenced by situational factors, it is also possible that the temporal scale sampled in the present study was not optimal for detecting underlying trait stability. The timescales over which personality‐related behaviors should remain stable in capuchins are not yet well established, and future longitudinal studies spanning a broader range of intervals may provide a more robust test of trait persistence.

Several of the associations between questionnaire ratings and behavioral observations were consistent with our predictions; however, others diverged or even contradicted expectations, and none confirmed previous associations reported by Morton et al. (2013). For example, the expectation of a positive correlation between Openness and Physical exploration was confirmed, as was the positive correlation between Neuroticism and Vocalization. On the other hand, the negative associations between Scratch and Openness and Sociability were not anticipated, and the correlation between Attentiveness and Social was expected to be positive, not negative. For Novelty Testing, none of the predictions could be confirmed. In total, out of 39 expected associations, only 4 were found consistent with expectations, 2 were found opposite to expectations, and 3 were found without any expectations. The scarcity of significant associations raises important conceptual and methodological questions. In this sample, the results suggest that the assumption of strong trait–behavior mapping may be overly simplistic, particularly in species with high behavioral flexibility such as capuchins. The absence of associations between novelty test data and questionnaire data may indicate that the novelty tests mostly capture situational reactivity. However, it may also reflect the limited temporal and contextual range of the assessment, which sampled behavior in a highly specific situation at only two time points. As such, the absence of convergence should not necessarily be interpreted as evidence that novelty responses lack a personality component. Collectively, these patterns imply that personality in capuchins may be multidimensional (Uher et al. 2013) and that expecting high cross‐method agreement may not be realistic when assessments differ substantially in both their temporal and methodological approach.

Similar challenges arise in human infant temperament research, where personality is assessed using parent reports, home observations, or structured laboratory tasks (Rothbart and Goldsmith 1985). Each approach offers unique advantages and constraints—not only in terms of practical feasibility but also in how personality is conceptualized. Parent reports provide broad, context‐integrated impressions but may be influenced by parental bias or expectations, while home observations capture behavior in naturalistic settings yet lack experimental control. Laboratory assessments allow for standardized elicitation of responses but may reflect situational reactivity rather than enduring traits (Stifter et al. 2008). These methodological differences often lead to low convergence across measures: correlations between parent ratings and observational data are typically modest (Seifer et al. 1994; Stifter et al. 2008). To address this issue, infant temperament research increasingly adopts multi‐method approaches, treating each measure as capturing complementary aspects of personality rather than expecting full agreement. Integrative models emphasize latent constructs derived from multiple indicators, longitudinal designs to assess stability, and context‐sensitive interpretations that acknowledge the dynamic nature of early temperament (Rothbart 2011). This perspective reframes divergence not as methodological failure but as evidence of multidimensionality—a principle that may also be relevant for nonhuman primate personality research.

The lack of replication of Morton et al.'s (2013) findings highlights the potential influence of contextual factors on trait expression. In the current study, housing condition emerged as a significant source of variation in capuchin monkeys' personality expression. It is unclear whether the causal factor for this variation relates to enclosure size, social group composition (i.e., pair housing vs. group housing), or other environmental factors that may have differed between groups, but it is clear that housing condition was associated with behavioral differences: pair‐housed capuchins were rated as more neurotic, less attentive, and displayed higher frequencies of stereotypic behaviors such as pacing and self‐grooming. Rather than representing a simple methodological confound, these results suggest that social and environmental conditions may shape how underlying personality traits are expressed. Differences in housing arrangements could limit opportunities for certain behaviors, alter behavioral tendencies, or both. Consequently, variation in housing should be considered when comparing personality structures across studies, as it may represent a meaningful source of behavioral variation. Similar patterns have been observed in other primates: Šlipogor et al. (2021) found that while common marmoset personality structures were temporally stable in captivity, trait expression varied with ecological and social context. Individuals that transitioned to breeder status became bolder, and wild marmosets exhibited either expanded or suppressed behavioral repertoires compared to captive counterparts, likely reflecting differences in environmental complexity and social dynamics. Such findings reinforce that personality is robust yet flexible, and that environmental and social factors can significantly shape behavioral expression. In contrast, sex and age effects in the current study were minimal, with no consistent patterns across methods. However, the absence of strong age‐related effects may reflect limitations of between‐subject designs rather than true developmental stability. Overall, these findings highlight the importance of considering housing conditions and adopting longitudinal designs to disentangle intrinsic traits from environmental influences.

More broadly, the findings highlight the need to distinguish personality structure from personality expression and to consider the temporal resolution and context of different assessment methods (Uher et al. 2013; Del Giudice 2022). Questionnaire ratings may integrate impressions accumulated across extended periods and situations, whereas naturalistic observations sample a narrower but ecologically familiar context, and experimental tests capture responses to standardized but brief events (Vazire et al. 2007; Uher and Visalberghi 2016). Divergence among these approaches may therefore reflect differences in sampling scope as well as differences in the constructs measured (Freeman et al. 2011; Tkaczynski et al. 2019). Future studies should explicitly evaluate how estimates of repeatability and cross‐method convergence change as observations are accumulated across sessions, contexts, and developmental periods (Dingemanse and Dochtermann 2013; Stamps and Groothuis 2010). Species‐specific work using repeated experimental trials, longer observational windows, multiple ecologically relevant contexts, and independent replication would help establish how much sampling is needed before stable dispositions can be distinguished from short‐term fluctuations (Bell et al. 2009; Takola et al. 2021). In captive studies, environmental and management variables such as enclosure characteristics and social‐group composition should be recorded and modeled as potential influences on personality expression, rather than treated solely as nuisance covariates (Rawlings et al. 2020; Šlipogor et al. 2021).

Overall, our findings add to a growing body of evidence suggesting that personality may be best understood as a context‐sensitive and multidimensional construct that is not fully captured by any single assessment method. Differences between methods may reflect not only the traits being measured but also the temporal and contextual scope of the observations on which they are based. Future work may therefore benefit from placing greater emphasis on integrating information across methods, timescales, and social environments. Such approaches may provide a more complete understanding of how stable dispositions and situational factors interact to shape behavioral expression.

## Author Contributions

**Annika Paukner:** conceptualization, investigation, writing – review and editing, writing – original draft, project administration, supervision. **Lauren J. Wooddell:** conceptualization, investigation, writing – review and editing. **Aurelie Jolivald:** writing – original draft, formal analysis, writing – review and editing, data curation, visualization.

## Supporting information


**Table S1:** Summary table of all analyses performed.
**Table S2:** Impacts of housing condition (group vs. paired), age category (adult vs. juvenile) and sex (female vs. male) on the frequency or total time spent expressing 9 behaviours during behavioural observations, modelled using multivariable Markov Chain Monte Carlo linear mixed models. All response variables and therefore coefficients are scaled. Values used as default in the model for each variable are in italics in the table caption. Significance is denoted as: for < 0.1, *for < 0.05, **for < 0.01, ***for < 0.001.
**Table S3:** Impacts of housing condition (breeding group vs. paired), age category (adult vs. juvenile), sex (female vs. male), year (2012 vs 2015), and test type (novel food vs. novel object vs. novel threat) on the frequency or total time spent expressing 7 behaviours during novelty tests, modelled using multivariable Markov Chain Monte Carlo linear mixed models. All response variables and therefore coefficients are scaled. Values used as default in the model for each variable are in italics in the table caption. Significance is denoted as: for < 0.1, *for < 0.05, **for < 0.01, ***for < 0.001.
**Table S4:** Impact of housing condition (breeding group vs. paired), age category (adult vs. juvenile), and sex (female vs. male) on personality questionnaire ratings, modelled using multivariable Markov Chain Monte Carlo linear mixed models. Response variables and therefore coefficients are scaled. Values used as default in the model for each variable are in italics in the table caption. Significance is denoted as: for < 0.1, *for < 0.05, **for < 0.01, ***for < 0.001.

## Data Availability

The data that support the findings of this study are available from the corresponding author upon reasonable request.
