# Supplementary material for: Personality in Capuchin Monkeys (Sapajus apella): Comparison of Assessment Methods and Constructs
Source: Am J Primatol. 2026 Oct 6;88(10):e70214. doi: 10.1002/ajp.70214 (PMC13639703; doi:10.1002/ajp.70214)
Supplement: Supplementary file 1 [file AJP-88-e70214-s001.docx]

**Supplementary Table 1.**

*Summary table of all analyses performed.*

| Model | Data | Response variable | Model | Fixed Effects | Analysis | Statistic derived |
| --- | --- | --- | --- | --- | --- | --- |
| mLMM_PF | All personality questionnaire ratings | { Assertiveness, Openness, Neuroticism, Sociability, Attentiveness } |  | - Age category - Sex - Housing condition | - Impact of age, sex, housing condition on score for personality factor_i_ | - Fixed effect coefficients |
| mLMM_BO | All Behavioural Observation data | {Self-Groom,  Head Toss,  Vocalisation,  Scratch,  Social,  Locomotion,  Visual Exploration, Physical Exploration, Pacing} | Null | N/A | - Temporal stability of behaviour during Behavioural Observation - Intercorrelations of behavioural expression during Behavioural Observation | - ICC - Unadjusted pairwise correlation coefficient between each pair of behaviours |
|  |  |  | Full | - Age category - Sex - Housing condition | - Impact of age, sex, housing condition on behaviour during Behavioural Observation - Temporal stability of behaviour during Behavioural Observation - Intercorrelations of behavioural expression during Behavioural Observation | - Fixed effect coefficients for each behaviour - Adjusted repeatability for each behaviour - Adjusted pairwise correlation coefficient between each pair of behaviours |
| mLMM_NT_2012 | Novelty Test data (Novel Food, Novel Object, Novel Threat) from 2012 | {Touch,  Latency,  Stationary, Locomotion,  Explore,  Scratch,  Threat} | Null | N/A | - Cross-context consistency of behavioural responses across novel contexts | - ICC for each behaviour |
|  |  |  | Full | - Age category - Sex - Housing condition - Test type (Food/Object/Threat) | - Cross-context consistency of behavioural responses across novel contexts | - Adjusted repeatability for each behaviour |
| mLMM_NT_2015 | Novelty Test data (Novel Food, Novel Object, Novel Threat) from 2015 | {Touch,  Latency,  Stationary, Locomotion,  Explore,  Scratch,  Threat} | Null | N/A | - Cross-context consistency of behavioural responses across novel contexts | - ICC for each behaviour |
|  |  |  | Full | - Age category - Sex - Housing condition - Test type (Food/Object/Threat) | - Cross-context consistency of behavioural responses across novel contexts | - Adjusted repeatability for each behaviour |
| mLMM_NT | Novelty Test data (Novel Food, Novel Object, Novel Threat) from 2012  Novelty Test data (Novel Food, Novel Object, Novel Threat) from 2015 | {Touch,  Latency,  Stationary, Locomotion,  Explore,  Scratch,  Threat} | Null | N/A | - Temporal stability of behaviour during Novelty Test - Intercorrelations of behavioural expression during Novelty Test | - ICC for each behaviour - Unadjusted pairwise correlation coefficient between each pair of behaviours |
|  |  |  | Full | - Age category - Sex - Housing condition - Test type (Food/Object/Threat) - Year (2012/2015) | - Impact of age, sex, housing condition on behaviour during Novelty Test - Temporal stability of behaviour during Novelty Test - Intercorrelations of behavioural expression during Novelty Test | - Fixed effect coefficients for each behaviour - Adjusted repeatability for each behaviour - Adjusted pairwise correlation coefficient between each pair of behaviours |
| mLMM_BOi_PFj | All Behavioural Observation data for behaviour_i_ + personality questionnaire ratings for Factor_j_ | {Behaviour_i_, Factor_j_}  where Behaviour_i_ is one of:   - Self-Groom - Head Toss - Vocalisation - Scratch - Social - Locomotion - Visual Exploration - Physical Exploration - Pacing   and Factor_j_ is one of:   - Assertiveness - Openness - Neuroticism - Sociability - Attentiveness |  | - Age category - Sex - Housing condition | - Correlations between behaviour during Behavioural Observation and personality scores | - Adjusted pairwise correlation coefficient between repeated measures of Behaviour_i_ and Personality Factor_j_ |
| mLMM_NTi_PFj | All Novelty Test data across years and contexts for behaviour_i_ + personality questionnaire ratings for Factor_j_ | {Behaviour_i_, Factor_j_}  where Behaviour_i_ is one of:   - Touch - Latency - Stationary - Locomotion - Explore - Scratch - Threat   and Factor_j_ is one of:   - Assertiveness - Openness - Neuroticism - Sociability - Attentiveness |  | - Age category - Sex - Housing condition   Applied to Behaviour_i_ only:   - Test type (Food/Object/Threat) - Year (2012/2015) | - Correlations between behaviour during Novelty Test and personality scores | - Adjusted pairwise correlation coefficient between repeated measures of Behaviour_i_ and Personality Factor_j_ |

**Supplementary Table 2.**

Impacts of housing condition (*group* vs. paired), age category (*adult* vs. juvenile) and sex (*female* vs. male) on the frequency or total time spent expressing 9 behaviours during behavioural observations, modelled using multivariable Markov Chain Monte Carlo linear mixed models. All response variables and therefore coefficients are scaled. Values used as default in the model for each variable are in *italics* in the table caption. Significance is denoted as: . for < 0.1, * for < 0.05, ** for < 0.01, *** for < 0.001.

| **Variable** | **Fixed Effect** | **Posterior Mean** | **Lower 95CI** | **Upper 95CI** | **Effective Sample** | **pMCMC** | **Sig** |
| --- | --- | --- | --- | --- | --- | --- | --- |
| **Head Toss** | Intercept | -0.369 | -1.098 | 0.331 | 3924.302 | 0.282 |  |
|  | Age: Juvenile | -0.034 | -1.416 | 1.499 | 3544.011 | 0.96 |  |
|  | Housing: Pair | 1.243 | -0.261 | 2.734 | 4000 | 0.099 | . |
|  | Sex: Male | -0.036 | -1.421 | 1.398 | 4000 | 0.949 |  |
| **Locomotion** | Intercept | 0.45 | 0.071 | 0.826 | 4000 | 0.022 | * |
|  | Age: Juvenile | -0.188 | -0.925 | 0.587 | 4000 | 0.601 |  |
|  | Housing: Pair | -0.465 | -1.282 | 0.376 | 4000 | 0.234 |  |
|  | Sex: Male | -0.437 | -1.161 | 0.307 | 3994.628 | 0.194 |  |
| **Pacing** | Intercept | -0.563 | -0.929 | -0.199 | 4039.135 | 0.006 | ** |
|  | Age: Juvenile | -0.103 | -0.878 | 0.611 | 3908.765 | 0.763 |  |
|  | Housing: Pair | 1.647 | 0.883 | 2.384 | 4000 | 0 | *** |
|  | Sex: Male | 0.105 | -0.589 | 0.818 | 4000 | 0.754 |  |
| **Physical Exploration** | Intercept | 0.191 | -0.174 | 0.57 | 4000 | 0.292 |  |
|  | Age: Juvenile | 0.461 | -0.31 | 1.2 | 4667.232 | 0.196 |  |
|  | Housing: Pair | -0.42 | -1.194 | 0.394 | 4195.597 | 0.274 |  |
|  | Sex: Male | -0.27 | -0.985 | 0.43 | 4000 | 0.427 |  |
| **Scratch** | Intercept | 0.093 | -0.218 | 0.423 | 4000 | 0.552 |  |
|  | Age: Juvenile | 0.073 | -0.522 | 0.739 | 4000 | 0.796 |  |
|  | Housing: Pair | 0.523 | -0.14 | 1.146 | 4000 | 0.107 |  |
|  | Sex: Male | -0.464 | -1.028 | 0.124 | 3683.481 | 0.112 |  |
| **Self-Groom** | Intercept | -0.144 | -0.493 | 0.181 | 4000 | 0.383 |  |
|  | Age: Juvenile | 0.27 | -0.396 | 0.929 | 4000 | 0.392 |  |
|  | Housing: Pair | 0.764 | 0.027 | 1.435 | 4000 | 0.034 | * |
|  | Sex: Male | -0.274 | -0.93 | 0.308 | 4000 | 0.364 |  |
| **Social** | Intercept | 0.372 | -0.11 | 0.825 | 4000 | 0.113 |  |
|  | Age: Juvenile | -0.435 | -1.354 | 0.479 | 4000 | 0.298 |  |
|  | Housing: Pair | -1.111 | -2.06 | -0.204 | 4000 | 0.024 | * |
|  | Sex: Male | 0.141 | -0.693 | 0.996 | 4000 | 0.732 |  |
| **Visual Exploration** | Intercept | 0.198 | -0.236 | 0.67 | 4000 | 0.365 |  |
|  | Age: Juvenile | -0.715 | -1.669 | 0.135 | 4000 | 0.112 |  |
|  | Housing: Pair | -0.975 | -1.866 | -0.02 | 4000 | 0.044 | * |
|  | Sex: Male | 0.461 | -0.364 | 1.349 | 4453.057 | 0.253 |  |
| **Vocalisation** | Intercept | 0.011 | -0.312 | 0.35 | 4000 | 0.949 |  |
|  | Age: Juvenile | -0.693 | -1.329 | -0.056 | 4396.963 | 0.036 | * |
|  | Housing: Pair | -0.825 | -1.526 | -0.165 | 4000 | 0.02 | * |
|  | Sex: Male | 0.692 | 0.08 | 1.292 | 4285.216 | 0.031 | * |

**Supplementary Table 3.**

Impacts of housing condition (*breeding group* vs. paired), age category (*adult* vs. juvenile), sex (*female* vs. male), year (*2012* vs 2015), and test type (*novel food* vs. novel object vs. novel threat) on the frequency or total time spent expressing 7 behaviours during novelty tests, modelled using multivariable Markov Chain Monte Carlo linear mixed models. All response variables and therefore coefficients are scaled. Values used as default in the model for each variable are in *italics* in the table caption. Significance is denoted as: . for < 0.1, * for < 0.05, ** for < 0.01, *** for < 0.001.

| **Variable** | **Fixed_Effect** | **Posterior Mean** | **Lower 95CI** | **Upper 95CI** | **Effective Sample** | **pMCMC** | **Sig** |
| --- | --- | --- | --- | --- | --- | --- | --- |
| **Object Exploration** | Intercept | 0.318 | -0.095 | 0.751 | 4000 | 0.137 |  |
|  | Age: Juvenile | 0.002 | -0.657 | 0.637 | 4000 | 0.978 |  |
|  | Housing: Pair | 0.583 | 0.021 | 1.134 | 3789.089 | 0.048 | * |
|  | Sex: Male | 0.147 | -0.438 | 0.711 | 3993.222 | 0.609 |  |
|  | T: Novel Object | -0.889 | -1.233 | -0.513 | 3986.203 | 0 | *** |
|  | T: Novel Threat | -1.246 | -1.617 | -0.87 | 3648.531 | 0 | *** |
|  | Year: 2015 | 0.198 | -0.115 | 0.518 | 3983.773 | 0.209 |  |
| **Latency** | Intercept | -0.199 | -0.686 | 0.249 | 4000 | 0.374 |  |
|  | Age: Juvenile | 0.07 | -0.682 | 0.814 | 3607.669 | 0.839 |  |
|  | Housing: Pair | -0.176 | -0.783 | 0.429 | 4000 | 0.575 |  |
|  | Sex: Male | -0.299 | -0.947 | 0.349 | 4000 | 0.35 |  |
|  | T: Novel Object | 0.301 | -0.094 | 0.697 | 4000 | 0.128 |  |
|  | T: Novel Threat | 1.051 | 0.636 | 1.432 | 4000 | 0 | *** |
|  | Year: 2015 | -0.004 | -0.347 | 0.36 | 3531.224 | 0.997 |  |
| **Locomotion** | Intercept | 0.886 | 0.335 | 1.385 | 3658.774 | 0.003 | ** |
|  | Age: Juvenile | -0.217 | -0.849 | 0.474 | 4000 | 0.52 |  |
|  | Housing: Pair | -1.145 | -1.761 | -0.515 | 4000 | 0 | *** |
|  | Sex: Male | -0.044 | -0.766 | 0.774 | 4000 | 0.893 |  |
|  | T: Novel Object | 0.063 | -0.18 | 0.302 | 4000 | 0.597 |  |
|  | T: Novel Threat | -0.12 | -0.375 | 0.127 | 4000 | 0.342 |  |
|  | Year: 2015 | -0.823 | -1.047 | -0.613 | 4000 | 0 | *** |
| **Scratch** | Intercept | -0.414 | -1.01 | 0.201 | 4000 | 0.163 |  |
|  | Age: Juvenile | 0.026 | -0.856 | 0.91 | 4000 | 0.959 |  |
|  | Housing: Pair | 0.69 | -0.076 | 1.466 | 4000 | 0.073 | . |
|  | Sex: Male | -0.153 | -1.069 | 0.642 | 4000 | 0.74 |  |
|  | T: Novel Object | 0.5 | 0.139 | 0.886 | 4000 | 0.013 | * |
|  | T: Novel Threat | 0.361 | -0.01 | 0.742 | 4333.021 | 0.063 | . |
|  | Year: 2015 | -0.159 | -0.478 | 0.183 | 4000 | 0.354 |  |
| **Stationary** | Intercept | -0.565 | -0.997 | -0.099 | 4000 | 0.014 | * |
|  | Age: Juvenile | -0.302 | -1.022 | 0.377 | 4000 | 0.393 |  |
|  | Housing: Pair | -0.336 | -0.91 | 0.286 | 3816.077 | 0.272 |  |
|  | Sex: Male | 0.065 | -0.519 | 0.679 | 3515.053 | 0.825 |  |
|  | T: Novel Object | 0.943 | 0.54 | 1.327 | 3765.144 | 0 | *** |
|  | T: Novel Threat | 1.076 | 0.681 | 1.49 | 4000 | 0 | *** |
|  | Year: 2015 | 0.051 | -0.295 | 0.405 | 3979.281 | 0.766 |  |
| **Threat** | Intercept | -0.218 | -0.657 | 0.251 | 4000 | 0.334 |  |
|  | Age: Juvenile | 0.306 | -0.443 | 1.035 | 4000 | 0.405 |  |
|  | Housing: Pair | -0.101 | -0.704 | 0.517 | 3722.608 | 0.739 |  |
|  | Sex: Male | 0 | -0.572 | 0.62 | 4000 | 0.982 |  |
|  | T: Novel Object | -0.013 | -0.442 | 0.415 | 3547.373 | 0.952 |  |
|  | T: Novel Threat | 0.897 | 0.497 | 1.344 | 4000 | 0 | *** |
|  | Year: 2015 | -0.155 | -0.54 | 0.2 | 4000 | 0.42 |  |
| **Touch** | Intercept | -0.474 | -1.083 | 0.101 | 4000 | 0.099 | . |
|  | Age: Juvenile | 0.171 | -0.716 | 0.97 | 4000 | 0.675 |  |
|  | Housing: Pair | 0.588 | -0.141 | 1.319 | 4000 | 0.125 |  |
|  | Sex: Male | 0.163 | -0.713 | 1.001 | 4881.215 | 0.698 |  |
|  | T: Novel Object | 0.476 | 0.134 | 0.808 | 4000 | 0.008 | ** |
|  | T: Novel Threat | -0.153 | -0.514 | 0.178 | 4000 | 0.38 |  |
|  | Year: 2015 | 0.008 | -0.298 | 0.315 | 4000 | 0.95 |  |

**Supplementary Table 4.**

Impact of housing condition (*breeding group* vs. paired), age category (*adult* vs. juvenile), and sex (*female* vs. male) on personality questionnaire ratings, modelled using multivariable Markov Chain Monte Carlo linear mixed models. Response variables and therefore coefficients are scaled. Values used as default in the model for each variable are in *italics* in the table caption. Significance is denoted as: . for < 0.1, * for < 0.05, ** for < 0.01, *** for < 0.001.

| Variable | Fixed Effect | Posterior Mean | Lower 95CI | Upper 95CI | Effective Sample | pMCMC | Sig |
| --- | --- | --- | --- | --- | --- | --- | --- |
| Assertiveness | Intercept | 0.084 | -0.737 | 0.85 | 4000 | 0.804 |  |
|  | Age: Juvenile | -1.381 | -2.908 | 0.18 | 3882.046 | 0.081 | . |
|  | Housing: Pair | -0.687 | -2.338 | 0.965 | 4000 | 0.388 |  |
|  | Sex: Male | 0.765 | -0.663 | 2.319 | 4000 | 0.296 |  |
| Openness | Intercept | -0.668 | -1.353 | 0.012 | 3949.438 | 0.057 | . |
|  | Age: Juvenile | 1.307 | -0.046 | 2.648 | 4000 | 0.062 | . |
|  | Housing: Pair | 0.521 | -0.871 | 1.945 | 4000 | 0.438 |  |
|  | Sex: Male | 0.346 | -0.904 | 1.725 | 4000 | 0.57 |  |
| Neuroticism | Intercept | -0.663 | -1.235 | -0.023 | 4000 | 0.030 | * |
|  | Age: Juvenile | 0.388 | -0.783 | 1.529 | 4000 | 0.492 |  |
|  | Housing: Pair | 1.581 | 0.33 | 2.829 | 4000 | 0.019 | * |
|  | Sex: Male | 0.115 | -1.005 | 1.242 | 4000 | 0.825 |  |
| Sociability | Intercept | 0.406 | -0.248 | 1.065 | 4000 | 0.212 |  |
|  | Age: Juvenile | 0.492 | -0.884 | 1.705 | 4208.785 | 0.437 |  |
|  | Housing: Pair | -1.021 | -2.545 | 0.259 | 4000 | 0.136 |  |
|  | Sex: Male | -0.322 | -1.565 | 0.925 | 4000 | 0.587 |  |
| Attentiveness | Intercept | 0.497 | 0.002 | 0.985 | 4000 | 0.044 | * |
|  | Age: Juvenile | 0.367 | -0.556 | 1.359 | 4000 | 0.438 |  |
|  | Housing: Pair | -1.497 | -2.532 | -0.479 | 4000 | 0.010 | ** |
|  | Sex: Male | -0.173 | -1.091 | 0.794 | 4000 | 0.694 |  |
|  |  |  |  |  |  |  |  |
